# A low-calorie rebaudioside D–glucose–sodium sweetener matches sucrose in palatability and neural responses in a functional MRI study of healthy adults

**DOI:** 10.3389/fnut.2025.1699670

**Published:** 2026-01-07

**Authors:** Yuko Nakamura, Rika Takahashi, Tadahiro Ohkuri

**Affiliations:** 1 Graduate School of Art and Sciences, Center for Evolutionary Cognitive Sciences, The University of Tokyo, Tokyo, Japan; 2 University of Tokyo Institute for Diversity and Adaptation of Human Mind (UTIDAHM), Tokyo, Japan; 3 Suntory Beverage and Food Limited, Kanagawa, Japan

**Keywords:** non-nutritive sweeteners, functional MRI, stevia, rebaudioside D, palatability

## Abstract

**Introduction:**

Non-nutritive sweeteners (NNSs) often taste less palatable than glucose-containing sugars, potentially because they differentially engage sweet taste pathways. In this study, we examined a low-calorie rebaudioside D–glucose–sodium (RebD mix) sweetener, formulated on the basis of previous evidence that its components can engage type 1 taste receptors 2 and 3 (T1r2/T1r3) and sodium–glucose cotransporter 1 (SGLT1)–related pathways. We tested whether the RebD mix matches sucrose in palatability and neural responses.

**Methods:**

A within-subject fMRI study was performed on 28 healthy adults [mean age 22.36 ± standard deviation (SD) 5.20 years; male = 22: body mass index (BMI) kg/m^2^ = 20.34 ± 2.22 SD]. During scanning, participants received three gustatory conditions in randomized order, with tasteless rinses (9 s) between trials: sucrose (234 mM), RebD mix (29.2 mM sucrose + 194.3 mM glucose + 0.156 mM RebD + 5 mM sodium gluconate), and RebD mix without sodium (Na^+^) (29.2 mM sucrose + 194.3 mM glucose + 0.184 mM RebD), which is known to enhance SGLT1 activation. Liking and wanting ratings for gustatory stimuli were obtained at scanning. Imaging analyses focused on the orbitofrontal cortex (OFC), a key region associated with palatability, and OFC-centered functional connectivity, using within-participant contrasts across the three gustatory conditions.

**Results:**

Liking and wanting did not differ across the three conditions. OFC response to the RebD mix did not differ from sucrose, whereas the RebD without Na^+^ condition showed greater OFC–postcentral gyrus connectivity than the other two conditions (ps_Bonferroni–corrected_ < 0.001). Across participants, OFC–postcentral connectivity in the RebD without Na+ condition was negatively associated with fullness measured at scanning.

**Conclusion:**

The RebD mix matched sucrose in subjective palatability and OFC responses, while removal of Na^+^ altered OFC– postcentral gyrus connectivity linked to satiety. These findings indicate that the RebD mix, a novel low-calorie formulation, may achieve sucrose-comparable palatability.

## 1 Introduction

The global prevalence of overweight and obesity has been rapidly increasing, with prevalence nearly doubling between 1990 and 2021 (1). The global increase in obesity and obese-related noncommunicable diseases, including metabolic disorders (e.g., type 2 diabetes mellitus), cardiovascular diseases, and some cancers, may be closely associated with the widespread consumption of sugar-sweetened beverages (SSBs), which are a major source of added sugars in the diet (2, 3). Given these public health concerns, non-nutritive sweeteners (NNSs) have emerged as popular alternatives to sugar, aiming to provide sweetness with minimal caloric impact. However, the low palatability of NNSs would impede their widespread utilization. For example, sucralose in Splenda^®^ (Heartland Food Products Group, LLC., Indiana, USA), one of the most widely used NNSs, has a bitter, metallic, and chemical taste compared to sucrose (4). Furthermore, brain imaging studies have indicated that, compare to sugar-sweetened solutions, NNSs would elicit smaller responses in brain regions including the insula/operculum, orbitofrontal cortex (OFC) and striatum (5). Moreover, brain responses to a NNS solution that was matched to the sweetness level of sucrose differed significantly from brain responses to the sucrose solution (6). Therefore, even if the sweetness level of NNSs is matched to sucrose, these NNSs would elicit different brain responses in regions associated with palatability than sucrose, and thus would be less palatable.

More recently, in addition to artificial sweeteners such as the sucralose, naturally occurring NNSs like stevia has been introduced into the market (7). Steviol glycosides, which are the sweet-tasting compounds found in the plant *stevia Rebaudiana*, are perceived as 200–300 times sweeter than sucrose (8, 9). Steviol glycosides are often perceived by consumers as a healthier alternative due to its plant-derived origin (10). Moreover, in comparison with the 2-week glucose intake trial, sucralose or saccharin intake for 2 weeks impaired glucose tolerance, whereas stevia intake did not (11). Therefore, steviol glycosides are less likely than other artificial NNSs to disturb sugar metabolism.

Among the steviol glycosides, rebaudioside D (RebD) is more palatable due to its lower bitterness compared to other steviol glycosides, such as rebaudioside A (12, 13). Thus, RebD is a potential candidate for better substitute for sugar than other NNSs. Additionally, recent studies have revealed differences in sweet taste receptors between glucose-containing sugars, such as sucrose, and NNSs (14, 15). Therefore, adding components to RebD that stimulate glucose-specific receptors could improve the sweetness of the RebD mixture, making it comparable to sucrose. It is well established that sweet taste is mediated primarily by the type 1 taste receptors 2 and 3 (T1r2/T1r3) on taste cells, which responds to glucose-containing sugars such as sucrose and glucose as well as NNSs (16, 17). More recently, sodium–glucose cotransporter 1 (SGLT1), a metabolic sugar sensor in the intestine (18), has also been shown to mediate T1r2/T1r3-independent detection of glucose-containing sugars in the oral cavity (19). SGLT1-related sweet taste detection is enhanced by adding Na^+^ (20), and this beneficial effect is observed for glucose-containing sugars but not for NNSs (15). Thus, glucose-containing sugars can co-activate T1r2/T1r3 and SGLT1, whereas most NNSs primarily engage T1r2/T1r3 alone. These receptor-level differences may lead to distinct brain responses and lower palatability for NNSs, and suggest that SGLT1-related sweet taste signals from the oral cavity may contribute to the detection of nutritive sugars. These findings suggested that combining RebD with components that influence SGLT1-related signaling might improve its sweet taste to be more comparable to sucrose. We therefore previously invented a new sweetener by combining RebD, glucose, and Na^+^ (the RebD mix), which achieved moderate sweetness while keeping the energy content of the mixture low (13). RebD itself is a non-nutritive sweetener, but the RebD mix used in the present study combines RebD with sucrose and glucose and is therefore more appropriately described as a low-calorie sweetener rather than a strictly non-nutritive sweetener. Based on prior receptor assays showing that RebD can activate T1r2/T1r3 (12) and that glucose and Na^+^ modulate SGLT1-related signaling (12, 15–17, 19, 20), the RebD mix was formulated with the aim of engaging both T1r2/T1r3- and SGLT1-related pathways, although receptor activation was not directly examined in the present study. However, the palatability of the RebD mix compared to sucrose has not been well examined. Additionally, it is unclear whether the brain response to the RebD mix would be similar to its response to glucose-containing sugars, such as sucrose.

In this study, we therefore aimed to compare the brain response to the RebD mix, the RebD mix without Na^+^, and sucrose, specifically in the OFC. The OFC is considered a secondary gustatory cortex (21), plays a broad role in processing gustatory pleasure—including hunger, attention, motivation, reward, and pleasantness (22)—and is strongly associated with the palatability of gustatory stimuli (23). It was hypothesized that the RebD mix would elicit OFC responses similar to those evoked by sucrose, whereas the RebD mix without Na^+^ might show different OFC responses because it lacks the Na^+^-dependent component that has been implicated in SGLT1-related signaling in previous studies (15, 20).

## 2 Materials and methods

### 2.1 Participants

A total of 28 participants were included in the current study [mean age 22.36 ± standard deviation (SD) 5.20 years; male = 22, female = 6; body mass index (BMI) kg/m^2^ = 20.34 ± 2.22 SD] (Table 1). Healthy individuals aged 18–45 years old are included. Individuals with a history of neurological injury; known genetic or medical disorders; food allergies; current or previous use of psychotropic medications; current or previous use of tobacco, cigarettes, or electronic cigarettes; or any exclusion criteria for MRI were excluded. The study procedures were approved by the Ethics Committee of the Department of Arts and Sciences at The University of Tokyo (Approval No. 955). Written informed consent was obtained from all participants.

**TABLE 1:** Demographics of participants and ratings for internal state at the functional MRI (fMRI) session.

	Age (years)	Sex (male/female)	Body mass index (kg/m^2^)	Fullness	Hunger
Mean ± S.D. Range	22.36 ± 5.20 18–42	22/6 –	20.34 ± 2.22 15.41–24.91	45.56 ± 20.63 13.62–90.40	37.97 ± 18.68 0.00–75.55

Range of hunger and fullness is 0–100. S.D., standard deviation.

### 2.2 fMRI session

**FIGURE 1:**
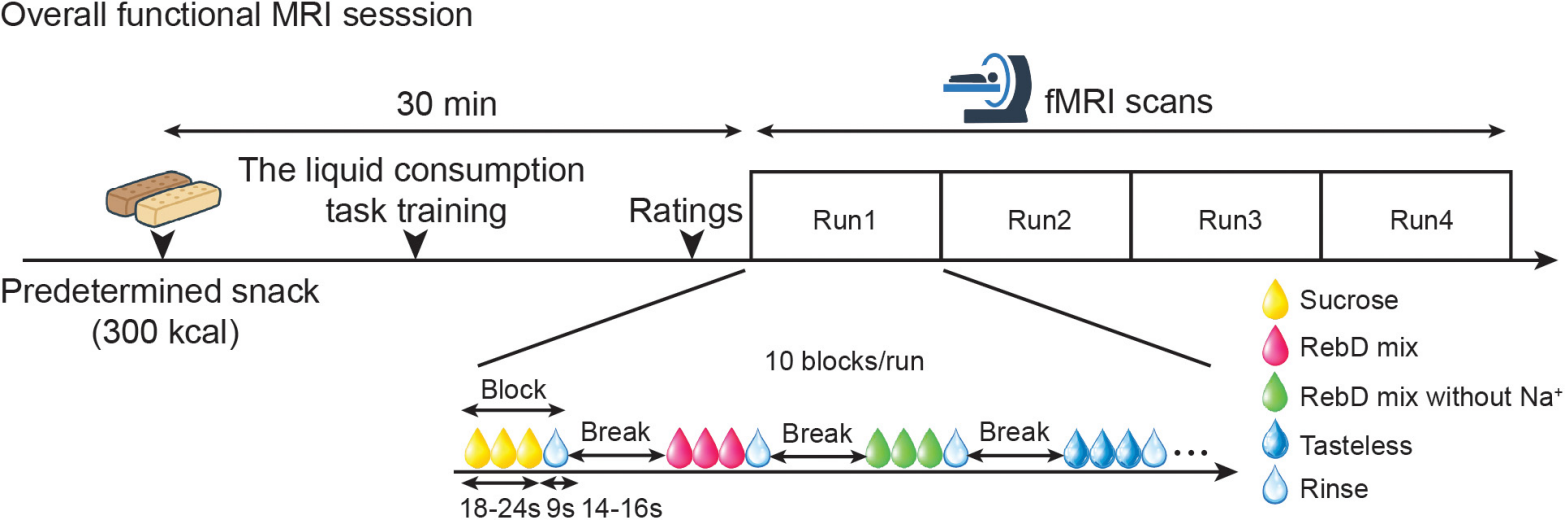
The experimental procedures. Participants were instructed to abstain from food and beverages, except for water, for at least three hours before visiting the laboratory. Anthropometric assessments were performed. Then, approximately 30 min before the fMRI scan, participants were instructed to consume the predetermined 300-kcal snack. Subsequently, practice for the liquid consumption task was performed. Immediately before the scan, they were asked to rate their internal state, as well as their liking, wanting, and intensity for each stimulus. Participants were then instructed to complete four runs of the liquid consumption task while being scanned.

On the day of the fMRI session, participants were instructed to abstain from food or beverages except water for a minimum of 3 h prior to their visit to the laboratory. Subsequent to their arrival, the subjects were measured for height and weight. These measurements were used to calculate the subjects’ BMI. Approximately 30 min before the fMRI scanning, the participants were instructed to consume the predetermined snack (300 kcal), with the objective of standardizing their internal state (hunger and fullness sensations). Following consumption of the snack, participants undertook a brief version (two blocks) of the liquid consumption task for the fMRI scan. Subsequently, the participants were escorted to the MRI room. Immediately before the fMRI scan, participants were instructed to rate their internal state (hunger and fullness) using a visual analog scale (VAS) ranging from 0 to 100. The left end of the scale was labeled “not at all” and the right end was labeled “more than ever.” A paired *t*-test showed there was no significant difference between hunger and fullness ratings (*p* = 0.25). We thus assumed that participants were neither extremely hungry nor extremely full at the time of the scan. Additionally, after consuming a small amount of each gustatory solution for the liquid consumption task, they rated their liking (“How much do you like this juice?”), wanting (“How much do you want to drink this juice?”), and intensity (“How thick is this juice?”) using the VAS that used for their internal state ratings (Table 2). Subsequently, participants were instructed to complete four runs of the liquid consumption task. However, two participants completed only two runs. Thus, a total of 26 participants completed four runs. The average length of one run was 7 min 16.8 ± 6.5 s (mean ± SD) (Figure 1).

**TABLE 2:** Ratings for liking, wanting, and intensity of each gustatory solution at the functional MRI (fMRI) session.

Ratings		Sucrose	RebD mix	RebD mix without Na^+^	*P*-value*
Liking	Mean ± S.E. Range	56.21 ± 3.61 21.53–83.46	55.54 ± 4.03 8.11–87.29	57.44 ± 2.62 31.84–91.89	0.937
Wanting	Mean ± S.E. Range	54.56 ± 3.55 22.57–96.11	53.11 ± 4.03 23.74–86.71	49.88 ± 3.58 16.02–86.64	0.895
Intensity	Mean ± S.E. Range	51.79 ± 3.43 7.39–80.61	50.43 ± 3.85 0.00–89.95	49.14 ± 3.35 21.73–89.36	0.063

Range of each rating is 0–100. S.E., standard error. *P-value of the Friedman test.

#### 2.2.1 Solutions for the liquid consumption task at the fMRI session

For the liquid consumption task, sucrose solution (234 mM sucrose, 32 kcal/100 ml), the RebD mix solution (29.2 mM sucrose + 194.3 mM glucose + 0.156 mM RebD + 5 mM sodium gluconate, 18 kcal/100 ml), and the RebD mix without Na^+^ solution (29.2 mM sucrose + 194.3 mM glucose + 0.184 mM RebD, 18 kcal/100 ml) were used as gustatory solutions. During the fMRI task, the total energy delivered was 17.92 kcal for sucrose and 10.08 kcal for both RebD mix solutions. To match the intensity of sweetness, the RebD mix included a smaller amount of RebD compared to the RebD mix without Na^+^, as Na^+^ enhances sweetness when ^mixedwithglucose.^ ^Inadditiontothese^ solutions, a tasteless solution was used as a control. For the tasteless solution, first, four tasteless solutions were prepared, including the original tasteless solution (25 mM KCl + 2.5 mM NaHCO_3_) and solutions with 25%, 50%, and 75% of the original concentration. Participants tasted a small amount of each solution and selected the one that tasted “the most like nothing” as their tasteless solution.

#### 2.2.2 Solution delivery

The timing and amount of solution delivered were controlled using programmable syringe pumps (NE-1000 One Channel Syringe Pump, NEW ERA PUMP SYSTEMS Inc., NY, USA). Details of the solution delivery system can be found elsewhere (24). In brief, through a clear polyvinyl chloride plastic tube, the syringe pumps delivered 0.80 mL of each solution over 2 s following a 1 s break.

#### 2.2.3 The liquid consumption task

For the fMRI session, participants performed four runs of the liquid consumption task. In this task, gustatory and tasteless solutions were delivered to a participant through a mouthpiece placed between the participant’s upper and lower lips.

Each solution was delivered randomly in ten blocks over four runs. Two or three blocks of each solution were performed in random order within a run. Within a block, a solution was delivered six to eight times (18–24 s), followed by three rinses (9 s). A tasteless solution was used for rinse, and the next block commenced after a short break (14–16 s) (Figure 1).

#### 2.2.4 MR image acquisition

All images were collected using a MAGNETOM Prisma 3.0 Tesla scanner with a 64-channel head coil (Siemens Healthineers, Erlangen, Germany). All high-resolution structural images of the brain were collected using a T1-weighted 3D MPRAGE protocol (repetition time (TR) = 1,900 ms, echo time (TE) = 2.53 ms, flip angle = 9°, field-of-view (FOV) = 256 × 256 mm^2^, resolution 1.0 × 1.0 × 1.0 mm^3^). As functional images, T2*-weighted images reflecting blood-oxygen-level-dependent (BOLD) signals were acquired using 2D gradient-echo echo-planar imaging (EPI) with an isotropic resolution (3.0 × 3.0 × 3.0 mm^3^), parallel acquisition factor = 3, TR = 2,000 ms, TE = 25 ms, 39 slices, and flip angle = 80° in a 192 mm^2^ FOV, transverse slices with phase encoding in the *P* > > A direction. All the images were acquired in an interleaved manner.

### 2.3 Statistical analysis

All functional and anatomical data underwent preprocessing using Statistical Parametric Mapping 12 (SPM12) (25) software with the CONN functional connectivity toolbox (CONN, version 22a) (26, 27). Functional and anatomical data were preprocessed using a modular preprocessing pipeline (28) including slice timing correction, creation of voxel-displacement maps, realignment with susceptibility distortion correction using fieldmaps, outlier detection, indirect segmentation and MNI-space normalization, and smoothing. Temporal misalignment between different slices of the functional data (acquired in interleaved bottom-up order) was corrected following SPM slice-timing correction (STC) procedure (29, 30), using sinc temporal interpolation to resample each slice BOLD timeseries to a common mid-acquisition time. Functional data were realigned using SPM realign and unwarp procedure (31) integrating fieldmaps for susceptibility distortion correction, where all scans were coregistered to a reference image (first scan of the first session) using a least squares approach and a six parameter (rigid body) transformation, and resampled using b-spline interpolation (32) to simultaneously correct for motion, magnetic susceptibility geometric distortions, and their interaction. Potential outlier scans were identified using ART (33) as acquisitions with framewise displacement above 0.9 mm or global BOLD signal changes above five standard deviations (34), and a reference BOLD image was computed for each subject by averaging all scans, excluding outliers. Functional and anatomical data were coregistered and normalized into standard MNI space, segmented into gray matter, white matter, and cerebrospinal fluid (CSF) tissue classes, and resampled to 2 mm isotropic voxels following an indirect normalization procedure (35) using SPM unified segmentation and normalization algorithm (36, 37) with the default IXI-549 tissue probability map template. Last, functional data were smoothed using spatial convolution with a Gaussian kernel of 6 mm full width half maximum (FWHM).

Preprocessed functional images from a total of 28 participants were brought to the individual level (first-level). The condition-specific effects (gustatory, tasteless, and rinse) at each voxel were estimated using a general linear model (GLM). The canonical hemodynamic response function provided by SPM12 was used to model the responses to the events. In the time-series analysis, a high-pass filter (270 s) was included in the filtering matrix to remove low-frequency noise and slow drifts from the signal. Confounding regressors from the preprocessing stage were also included in the model as covariates of no interest. Then, the [sucrose > tasteless], [RebD mix > tasteless], and [RebD mix without Na^+^ > tasteless] contrast images were created for individual participants.

Each contrast image was used in the subsequent group level (second-level) analysis. To examine the effect of each gustatory condition, a repeated measure analysis of variance (ANOVA) was performed using a voxel-wise GLM including ratings for intensity as a covariate-of-no-interest since there was a difference in ratings for intensity at a trend revel (*p* = 0.063) across three gustatory conditions. The predicted effect of these analyses was tested using the region of interest (ROI) approach. The ROI was created based on the Neurosynth (38) meta-analytic functional map for the term “taste” (80 studies, downloaded 15 February 2025). First, the downloaded brain map was thresholded at 60 % of probability (greater than 60 % of the peak z-value), then masked with the anatomical mask of the OFC crated with the automatic anatomical labeling (AAL) atlas (39) using the WFU Pickatlas toolbox (40, 41). Then, the sphere with 6 mm radius centered on the peak voxel [(x, y, z) = (−21.8, 33.5, −16.7)] of the masked brain map was created as the ROI in the OFC. The unpredicted effect of these analyses was tested using whole brain analysis. The threshold was set at *p*_family–wise error rate (FWE)–corrected_ < 0.05 for the ROI and whole brain approaches, respectively.

Subsequently, whole-brain searchlight multivoxel pattern analysis (MVPA) (42) was employed to identify the brain regions that exhibited varied responses to each solution using the Decoding Toolbox (43) for 28 participants. This analysis was executed in accordance with a linear support vector machine approach. The training and testing of the analysis were conducted on individual-level regression coefficient maps derived from the individual level analysis, employing a leave-one-run-out cross-validation strategy. For this analysis, fMRI data was preprocessed using a modular preprocessing pipeline without MNI-space normalization and smoothing. Then, to generate the regression coefficient maps used as input for the MVPA, a separate individual-level regression model was applied to the fMRI data, which modeled each of the five conditions (sucrose, RebD mix, RebD mix without Na^+^, tasteless, and rinse) including confounding regressors. For each participant, a separate searchlight analysis was performed using a searchlight with a radius of three voxels, evaluating all three pairwise gustatory contrasts (sucrose vs. RebD mix, sucrose vs. RebD mix without Na^+^, RebD mix vs. RebD mix without Na^+^). The output of this searchlight analysis was a voxelwise map of average classification accuracy minus chance (33% for the multiclass comparisons). To evaluate the classification results at the group level, the resulting classification maps were warped to the standard MNI space, and applied a spatial smoothing (6 mm FWHM) to normalize the distribution of scores. Then, a group-level voxel-wise GLM was applied using permutation-based non-parametric testing with 5,000 permutations (44). Threshold-free cluster enhancement (TFCE) was employed to assess cluster significance (45), with threshold of *p*_FWE–corrected_ < 0.05.

Finally, for seed to whole brain functional connectivity analysis, the generalized psychophysiological interaction (gPPI) (46, 47) analysis was adapted using preprocessed functional images. Since two participants completed only two runs, these two participants were excluded from this analysis, and a total of 26 participants were included in the gPPI. For this, the ROI used for the ANOVA was adapted as a seed. In addition, a sphere with a radius of 6 mm centered on the peak voxel [(x, y, z) = (34, 18, 6)] of the results from the whole-brain searchlight MVPA was used as a seed. At the individual level, separately, for each pair of seed and target areas, a gPPI was defined with BOLD time-series extracted from the seed as physiological factors, boxcar signals characterizing each individual gustatory or taste condition convolved with an SPM canonical hemodynamic response function as psychological factors, and the product of the two as psychophysiological interaction terms. Functional connectivity changes across conditions were characterized by the multivariate regression coefficient of the psychophysiological interaction terms in each model. At the group level, a GLM was estimated with first-level connectivity maps as dependent variables and conditions (sucrose, RebD mix, RebD mix without Na^+^) as independent variables to test for differences in connectivity across conditions using *F*-test. Inferences were drawn at the level of individual clusters. Cluster-level inferences were based on parametric statistics from Gaussian Random Field theory (48). Results were thresholded using a combination of a cluster-forming *p* < 0.001 voxel-level threshold, and a *p*_false discovery rate (FDR)–corrected_ < 0.05 cluster-size threshold (49). Two seeds, the OFC and the insula, were used to create seed-to-whole brain connectivity maps separately. Thus, the significance level was set at *p*_FDR–corrected_ < 0.025 (0.05/2).

Previous studies have shown that SGLT1-related sweet taste signals from the oral cavity may play a role in detecting sugars (15, 20), and sweet taste rapidly inhibits hunger-promoting agouti-related peptide-expressing (AgRP) neurons in the brain (50). Therefore, differences in connectivity among the three gustatory conditions could be related to internal states. Thus, the correlations between the connectivity that showed a significant difference across the three conditions and the internal states were compared between conditions. First, correlations between hunger or fullness and connectivity, controlling for BMI as a potential confounder were calculated. For this, partial correlations with the ppcor package (51) in R (version 4.4.1, R Foundation for Statistical Computing, Vienna, Austria) was used. Each internal state was tested three times. Thus, statistical significance was set at α = 0.0167 (0.05/3). Then, to compare the strength of partial correlations, Steiger’s test (52) for dependent correlations using the cocor package (53) was employed. This test is specifically designed to compare correlations that share a common variable and accounts for the intercorrelations between predictor variables. Each correlation coefficient was tested twice. Thus, statistical significance was set at α = 0.025 (0.05/2).

## 3 Results

### 3.1 Participant characteristics and behavioral ratings

Participant characteristics and behavioral ratings (hunger, fullness, liking, wanting, and intensity for each gustatory condition) are summarized in Tables 1, 2. Exploratory comparisons between males (*n* = 22) and females (*n* = 6) revealed no sex differences in age (*p* = 0.69) or in ratings for internal states (hunger and fullness) (*ps* > 0.28). BMI was higher in males than in females (*p* = 0.038). Ratings of liking, wanting, or intensity for any of the gustatory stimuli did not significantly differ between males and females (*ps* > 0.26).

The Friedman test showed that liking, wanting, and intensity ratings for all gustatory stimuli were not significantly different (*ps* > 0.063) (Table 2).

### 3.2 Brain response to each gustatory condition

**FIGURE 2:**
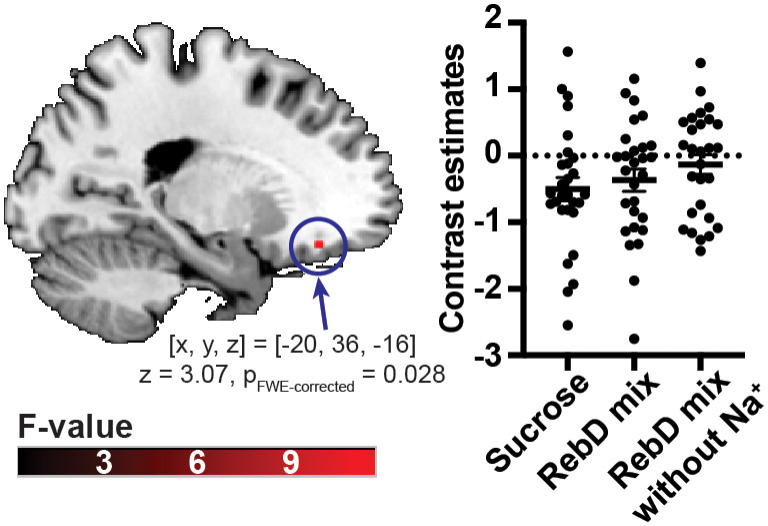
Averaged brain response to each gustatory solution. The scatter plot indicates the left orbitofrontal cortex response to each gustatory solution. The color bar indicates *F*-value.

There was significant averaged brain response to each gustatory contrast in the ROI [(x, y, z) = (−20, 36, −16), z = 3.07, *p*_FWE–corrected_ = 0.028, cluster size = 3 voxels], while there was no significant difference in brain response to each gustatory contrast (Figure 2). Since the sex distribution was not balanced well in this study, sex was included as a covariate in additional analyses. There was still significant averaged brain response to each gustatory contrast in the ROI [(x, y, z) = (−20, 36, −16), z = 3.12, *p*_FWE–corrected_ = 0.024, cluster size = 3 voxels]. The whole-brain analysis showed that there were no unexpected effects of gustatory contrasts.

### 3.3 Results of whole-brain searchlight MVPA

**FIGURE 3:**
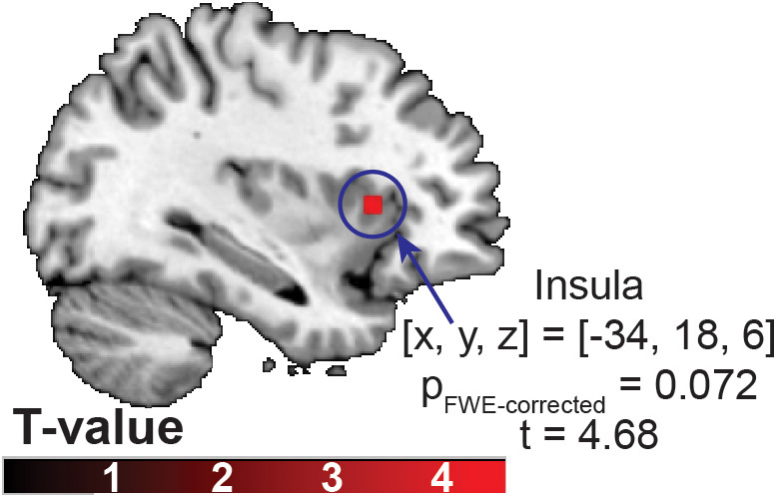
The brain region identified using a multivariate searchlight analysis, trained to distinguish between sucrose, the RebD mix, and the RebD mix without Na^+^ solutions. The color bar indicates T-value.

The whole-brain searchlight MVPA identified the brain region that exhibited significant, above chance classification accuracy for discriminating between gustatory conditions in the anterior insula at a trend level [(x, y, z) = (−34, 18, 6), p_FWE–corrected_ = 0.072, *t* = 4.68, an averaged accuracy at the peak voxel = 40.71%) (Figure 3).

### 3.4 Connectivity analysis with the gPPI

**FIGURE 4:**
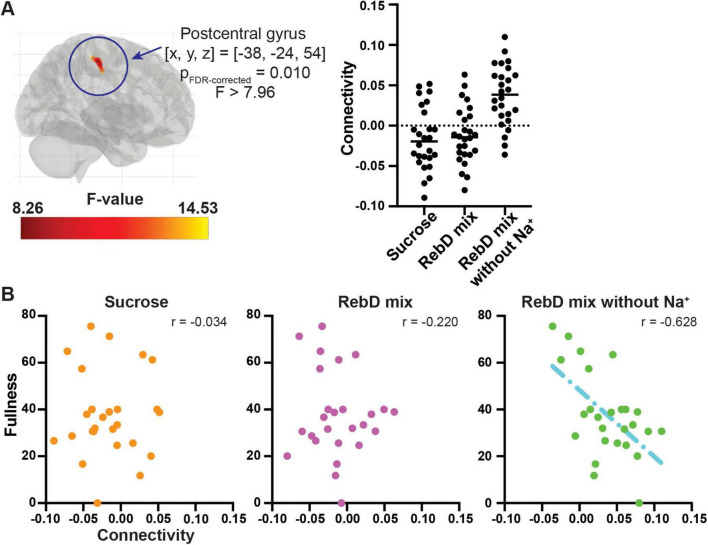
Results of the generalized psychophysiological interaction analysis. **(A)** The brain region that showed significantly different connectivity with the left orbitofrontal cortex across three distinct gustatory conditions. The scatter plot represents connectivity between the left orbitofrontal cortex and the postcentral gyrus in each gustatory condition. The color bar indicates *F*-value. **(B)** Associations between ratings for fullness and the left orbitofrontal cortex-the postcentral gyrus connectivity in each condition. The y-axis indicates ratings for fullness, and the x-axis indicates connectivity.

Firstly, an examination was conducted to detect any differences in functional connectivity with the seed areas (OFC or insula) across the three conditions. Functional connectivity between the left OFC and the left postcentral gyrus significantly differs across three conditions [(x, y, z) = (−38, −24, 54), *p*_FDR–corrected_ = 0.010, F(2, 50) > 7.96, cluster size = 62 voxels] (Figure 4A). As additional analysis, sex was included as a covariate. There was still significant difference in connectivity between the left OFC and the left postcentral gyrus across three conditions [(x, y, z) = (−38, −22, 50), *p*_FDR–corrected_ = 0.0024, F(2, 48) > 8.00, cluster size = 78 voxels]. There were no differences in insula-to-whole-brain connectivity maps across the three conditions.

*Post hoc* analysis using extracted connectivity values showed that the connectivity between the OFC and left postcentral gyrus in the RebD mix without Na^+^ condition was greater than that in the RebD mix or sucrose conditions (*ps*_Bonferroni–corrected_ < 0.001). The connectivity in the RebD mix condition did not significantly differ from that in the sucrose condition.

Partial correlations between fullness and connectivity measures, controlling for BMI, were as follows: connectivity in the sucrose condition (*r* = −0.034, *p* = 0.871), in the RebD mix condition (*r* = −0.220, *p* = 0.291), and in the RebD mix without Na^+^ (*r* = −0.628, *p* < 0.001). Thus, there was a significant negative association between connectivity in the RebD without Na^+^ and fullness. Hunger was not significantly correlated with any connectivity (*p* > 0.157). Steiger’s test revealed no significant difference in correlation strength between the sucrose and RebD mix conditions (*z* = 0.679, *p* = 0.500). However, there was a significant difference between the sucrose and RebD mix without Na^+^ conditions (*z* = 2.591, *p* = 0.001). Additionally, there was a trend toward a significant difference in correlation strength between the RebD mix and the RebD mix without Na^+^ conditions (*z* = 2.008, *p* = 0.045) (Figure 4B).

## 4 Discussion

The current study examined the brain response to the newly developed sweetener RebD mix, RebD mix without Na^+^, and sucrose in the OFC, which processes the palatability of gustatory stimuli (23). Additionally, the brain response to these sweeteners was examined throughout the entire brain, and the anterior insula was found to be associated with the decoding of these sweeteners. Functional connectivity analysis revealed significantly greater connectivity between the OFC and postcentral gyrus in the RebD without Na^+^ condition than in the other two gustatory conditions. Furthermore, this connectivity in the RebD without Na^+^ condition was negatively correlated with fullness measured at the fMRI scan.

There were no significant differences in OFC responses to each sweet solution, which corresponded to the ratings comparison results for liking, wanting, and intensity. However, there was a significant averaged brain response to each sweet solution in the OFC. Since liking or wanting ratings for all sweet solutions were nearly or greater than half of the rating range, those solutions would be equally palatable and represented by the OFC response, which could have a role in processing palatability of gustatory stimuli (23).

Multivoxel pattern analysis indicated that the anterior insula is related to discriminating between sweet taste solutions. This region of the insula is involved in taste detection (54) and taste discrimination (55). Thus, although participants were not instructed to distinguish between three different sweet solutions, differences in the composition of the sweet substances would automatically be reflected in the anterior insula, even if the participants did not recognize the differences.

Connectivity analysis revealed that connectivity between the OFC and postcentral gyrus was greater in the RebD mix without Na^+^ condition compared to other two gustatory conditions. The postcentral gyrus has been reported to be connected with impulsivity (56). In addition, the postcentral gyrus showed a significant response to marijuana cues in regular marijuana users who had abstained from use for 3 days (57). Furthermore, working for the preferred food resulted in a stronger response in the postcentral gyrus (58). Thus, connectivity between the OFC and the postcentral gyrus could be involved in cravings or motivation for food. Since the connectivity in the RebD mix without Na^+^ was greater than in the other two conditions, the SGLT1-related signal would reduce this connectivity, as the RebD mix and sucrose are expected to stimulate the SGLT1-related signal (15, 19). Additionally, this connectivity in the RebD mix without Na^+^ condition was negatively associated with fullness measured at the fMRI scan, while the connectivity in other two conditions were not significantly associated with fullness. Thus, sweet taste associated with SGLT1-related signals might mediate the OFC-postcentral gyrus connectivity to reduce cravings for sweet tastes.

Glucose-induced intestinal SGLT1 activation modulates the secretion of intestinal hormones that regulate glucose homeostasis, including glucagon-like peptide-1 (GLP-1) and glucose-dependent insulinotropic peptide (GIP) (59–61), which are well known as satiety hormones (62). Therefore, sweet taste associated with SGLT1-related signals may be linked to feelings of satiety. In addition, sweet taste rapidly inhibits hunger-promoting AgRP neurons in the brain, and the reduction in AgRP neuron activity was much larger following consumption of nutritive foods (e.g., sucrose) than their non-nutritive counterparts (e.g., sucralose) (50). In sum, SGLT1-related sweet taste signals from the oral cavity may play a role in detecting nutritive sugars (15, 20), and glucose-induced intestinal SGLT1 activation modulates satiety sensation (59–62), and nutritive sweet taste significantly reduces hunger-promoting neural activation (50). Therefore, SGLT1-related sweet taste could be related to satiety sensation, although there is no conclusive evidence indicating that the sweet taste sensation elicited by SGLT1 plays a role in satiety for sweet tastes. Unlike sucrose or other glucose-containing sugars, most NNSs primarily activate the T1r2/T1r3 sweet taste receptors and do not substantially engage SGLT1 (15–17). Consistent with this distinct sensory profile, a recent survey reported that 63.6% of participants perceived NNSs as sweet but not sugar-like and indicated that they would prefer them to taste more like sugar (63). Taken together with the above findings on SGLT1-related signaling, these observations suggest that targeting SGLT1-mediated sweet taste—for example, by enhancing SGLT1 activation through the addition of Na^+^—may provide a novel approach to developing lower-calorie sweeteners with a more desirable, sugar-like taste. Such lower-calorie, sugar-like sweeteners could support the reformulation of food products and may be beneficial for diabetes management and the prevention of excessive weight gain.

The current study has several limitations. First, the sample size would be relatively small. A previous study that performed a similar analysis included only 20 participants (55), and the median sample size for fMRI experiments with gustatory stimuli was 24 (64). Thus, the sample size of the current study would be acceptable. However, future studies should have a greater sample size. Second, the current study did not examine the brain response to the detailed characteristics of the sweet taste quality of the RebD mix, such as aftertaste or sense of viscosity. Future studies should examine differences in brain responses to the detailed characteristics of sweet taste quality between sucrose and the RebD mix to further understand the neural mechanisms of RebD mix perception. Third, this study included more males (*n* = 22) than females (*n* = 6). Therefore, the current results may be affected by sex, although exploratory analyses did not reveal sex differences in behavioral ratings, and including sex as a covariate did not significantly alter the imaging or connectivity results. Future studies with larger and more sex-balanced samples could further clarify the palatability of the RebD mix and the associated brain responses to this novel sweetener. Fourth, this study did not systematically assess alcohol consumption. Although participants were instructed to abstain from food and beverages except water for at least 3 h before scanning, possible effects of habitual alcohol use on palatability or neural responses cannot be entirely ruled out. Finally, another limitation is that we did not assess blood glucose or other metabolic responses to the gustatory solutions. Given the relatively small total energy delivered during scanning and the randomized presentation of conditions, large systematic effects of acute glycemic changes on the imaging results are unlikely; however, we did not directly measure blood glucose. Thus, we cannot make conclusions about glycemic effects.

The current study examined brain response to the RebD mix, the RebD mix without Na^+^, and sucrose. There were no significant differences in the ratings of liking, wanting, or intensity for those solutions or the OFC responses to them. However, the anterior insula response pattern differed across three solutions, reflecting differences in compounds of each sweetener. Additionally, connectivity analysis indicated that the RebD mix without Na^+^, rather than the RebD mix or sucrose, would be associated with reduced satiety for sweet tastes, as the OFC-postcentral gyrus connectivity in the RebD mix without Na^+^ condition was negatively correlated with fullness measured at the fMRI scan. This result would suggest that SGLT1 activation, enhanced by Na^+^, is important for satiety in response to sweet tastes. Overall, the RebD mix is a less nutritious sweetener, yet it would be comparable to sucrose with respect to palatability and satisfaction.

## Data Availability

The original contributions presented in this study are included in this article/supplementary material, further inquiries can be directed to the corresponding author.
